# HLA Class I Allele Expression and Clinical Outcome in De Novo Metastatic Prostate Cancer

**DOI:** 10.3390/cancers12061623

**Published:** 2020-06-18

**Authors:** Savvas Stokidis, Sotirios P. Fortis, Paraskevi Kogionou, Theodoros Anagnostou, Sonia A. Perez, Constantin N. Baxevanis

**Affiliations:** 1 Cancer Immunology and Immunotherapy Center, Saint Savas Cancer Hospital, 171 Alexandras avenue, 11522 Athens, Greece; savstok@gmail.com (S.S.); fortis@ciic.gr (S.P.F.); pkogionou@gmail.com (P.K.); perez@ciic.gr (S.A.P.); 2 Department of Urology, Saint Savas Cancer Hospital, 171 Alexandras avenue, 11522 Athens, Greece; theodoreanagnostou@gmail.com

**Keywords:** HLA-alleles, HLA-A*02:01, HLA-A*24:02, prognosis, de novo metastatic prostate cancer

## Abstract

The prognostic value of human leukocyte antigen (HLA) class I molecules in prostate cancer (PCa) remains unclear. Herein, we investigated the prognostic relevance of the most frequently expressed HLA-A alleles in Greece (A*02:01 and HLA-A*24:02) in de novo metastatic hormone-sensitive PCa (mPCa), which is a rare and aggressive disease characterized by a rapid progression to castration-resistance (CR) and poor overall survival (OS), contributing to almost 50% of PCa-related deaths. We identified 56 patients who had either progressed to CR (these patients were retrospectively analyzed for the time to the progression of CR and prospectively for OS) or had at least three months’ follow-up postdiagnosis without CR progression and, thus, were prospectively analyzed for both CR and OS. Patients expressing HLA-A*02:01 showed poor clinical outcomes vs. HLA-A*02:01^−^negative patients. HLA-A*24:02^−^positive patients progressed slower to CR and had increased OS. Homozygous HLA-A*02:01 patients progressed severely to CR, with very short OS. Multivariate analyses ascribed to both HLA alleles significant prognostic values for the time to progression (TTP) to CR and OS. The presence of HLA-A*02:01 and HLA-A*24:02 alleles in de novo mPCa patients are significantly and independently associated with unfavorable or favorable clinical outcomes, respectively, suggesting their possible prognostic relevance for treatment decision-making in the context of precision medicine.

## 1. Introduction

Prostate cancer (PCa) is the second-most common cancer in men [1] and the second leading cause of cancer-related deaths in men [2,3]. PCa incidence and mortality vary widely among countries and ethnic populations [4]. Most patients present with a localized disease at first diagnosis. However, approximately 4% of PCa patients have a metastatic disease at the time of diagnosis, and they are defined as de novo metastatic PCa patients [5,6]. De novo metastatic and hormone-sensitive prostate cancer (mHSPCa) represents a biologically aggressive disease characterized by poor prognosis [7,8]. De novo metastatic prostate cancer (mPCa) contributes to almost 50% of PCa-related deaths [9].

The heterogeneous prognosis of PCa underlines the necessity of developing prognostic/predictive biomarkers, paving the way for precision medicine for effective disease management [10]. Although established clinical parameters (e.g., prostate-specific antigen (PSA), Gleason score (GS), etc.) allow a certain risk stratification, they are still not sufficient for accurate clinical outcome predictions [11,12]. Until now, several prognostic models have been validated in metastatic castrate-resistant PCa [13,14,15] but not in mHSPCa and, particularly, de novo mPCa. In large phase III clinical trials investigating the efficacy of docetaxel or abiraterone plus androgen deprivation therapy in mHSPCa, two different prognostic scores were used to stratify high vs. low-risk patients [16]. However, there was a lack of complete concordance between classifications when comparing OS, highlighting the necessity for the identification of more factors for patient prognosis [17]. Although many factors have been already explored for their possible prognostic role, including the Gleason score (GS), performance status (PS), age and PSA levels at the time of diagnosis, and number and site of metastases, as well as the disease burden, none of them have been prospectively validated [5].

PCa, although described as an immunogenic disease, its immunogenicity is impeded by a broadly immunosuppressive microenvironment consisting of complex interactions between local immunosuppressive cells, including regulatory T cells, tumor-associated macrophages, and myeloid-derived suppressor cells, as well as cancer cells that perform collaborative interactions to downregulate antitumor immune responses, thereby promoting disease progression [18,19]. Master regulators of these complex processes are human leukocyte antigen (HLA) alleles, which genetically restrict the priming, as well as the effector phase, of T cells in the process of the development of antitumor immune interactions [20,21]. As a consequence, HLA-alleles have been related to the prevalence or outcome of several diseases as autoimmunity and cancer [22]. HLA-A*02 has been shown to be overrepresented among patients with ovarian and PCa and to be associated with higher mortality rates [23], whereas its expression in lung adenocarcinoma and epithelial ovarian cancer patients has been associated with an unfavorable prognosis. In contrast to the unfavorable role of HLA-A2, in a recent phase I trial from our group, we noticed that, among PCa patients vaccinated with a CD4+ T cell-stimulating HER-2/neu hybrid-peptide, the HLA-A*24 allele expression conferred a better clinical outcome [24,25]. 

In the current retrospective/prospective study, we investigated the prognostic relevance of the most common HLA-A* alleles in Greece, HLA-A*02:01 and HLA-A*24:02 [26], in de novo mPCa patients following the standard-of-care treatments according to their disease statuses. Their possible prognostic potentials could aid in the decision-making process contributing to timely and right treatment decisions, avoiding possible under- or overtreatments.

## 2. Results

### 2.1. HLA-A*02:01 and HLA-A*24:02 Alleles Influence the Progression to CR in De Novo mPCa Patients

**Figure 1:**
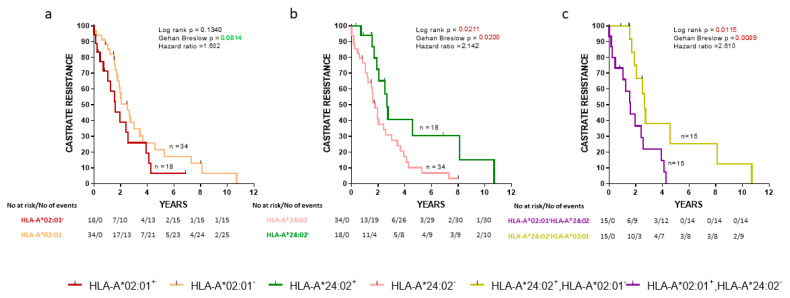
Kaplan-Meier curves illustrate the time to castration-resistance (CR) progression among groups of de novo metastatic prostate cancer (mPCa) patients. (**a**) HLA-A*02:01^+^ vs HLA-A*02:01^−^; (**b**) HLA-A*24:02^+^ vs HLA-A*24:02^−^; (**c**) HLA-A*02:01^+^ HLA-A*24:02^−^ vs HLA-A*24:02^+^ HLA-A*02:01^−^. Shown are statistical differences and hazard ratios among the groups.

Initially, we evaluated the association between the progression to CR and expression of HLA alleles in the cohort of our de novo mPCa patients. As shown in Figure 1a, HLA-A*02:01^−^ patients exhibited a trend for a longer time to CR than HLA-A*02:01^+^ patients. In addition, HLA-A*24:02 expression was also associated with a strong statistically significant delayed appearance of CR (Figure 1b) (*p* = 0.0211). Only a very limited number of patients (*n* = 3) coexpressed HLA-A*02:01 and HLA-A*24:02, and therefore, this group was excluded from further analyses. Notwithstanding, this interesting patient group will be the target of our future investigations to see if and to what extent the presence of the one allele might counteract the effects of the other. Thus, we continued our analyses with CR as an endpoint with patients expressing the one or the other allele. As expected, HLA-A*24:02^+^HLA-A*02:01^−^ patients showed a statistically significant longer time to CR than HLA-A*24:02^−^HLA-A*02:01^+^ patients (*p* = 0.0115) (Figure 1c).

### 2.2. HLA-A*02:01 and HLA-A*24:02 Alleles Influence OS in De Novo mPCa Patients

**Figure 2:**
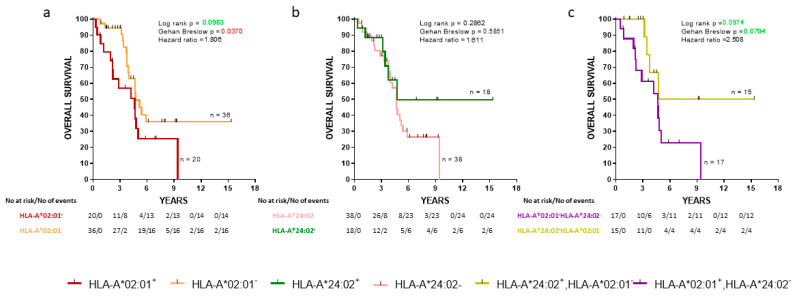
Kaplan-Meier curves illustrate the time to the overall survival (OS) progression among groups of de novo mPCa patients. (**a**) HLA-A*02:01^+^ vs HLA-A*202:01^−^; (**b**) HLA-A*24:02^+^ vs HLA-A*24:02^−^; (**c**) HLA-A*02:01^+^ HLA-A*24:02^−^ vs HLA-A*24:02^+^ HLA-A*202:01^−^. Shown are statistical differences and hazard ratios among the groups.

We also analyzed the same groups of de novo mPCa patients with the overall survival (OS) as an endpoint. The HLA-A*202:01^−^cohort exhibited a statistically significant decrease of early death (Gehan-Breslow, *p* = 0.0370) compared to the HLA-A*02:01 expressors (Figure 2a). No statistically significant difference in OS between HLA-A*24:02^+^ patients and HLA-A*24:02^−^ was observed (Figure 2b). Additionally, patients expressing the HLA-A*24:02 allele but not HLA-A*02:01 were compared with patients having the reverse combination. HLA-A*24:02^+^HLA-A*202:01^−^ patients showed a strong trend for improved OS than HLA-A*24:02−HLA-A*02:01^+^ patients (zero vs. seven deaths at four years postdiagnosis, respectively; Gehan-Breslow, *p* = 0.0794) (Figure 2c).

### 2.3. The Prognostic Value of HLA-A*02:01 and HLA-A*24:02 Alleles Is Independent of Established Prognostic Clinicopathological Factors

**Figure 3:**
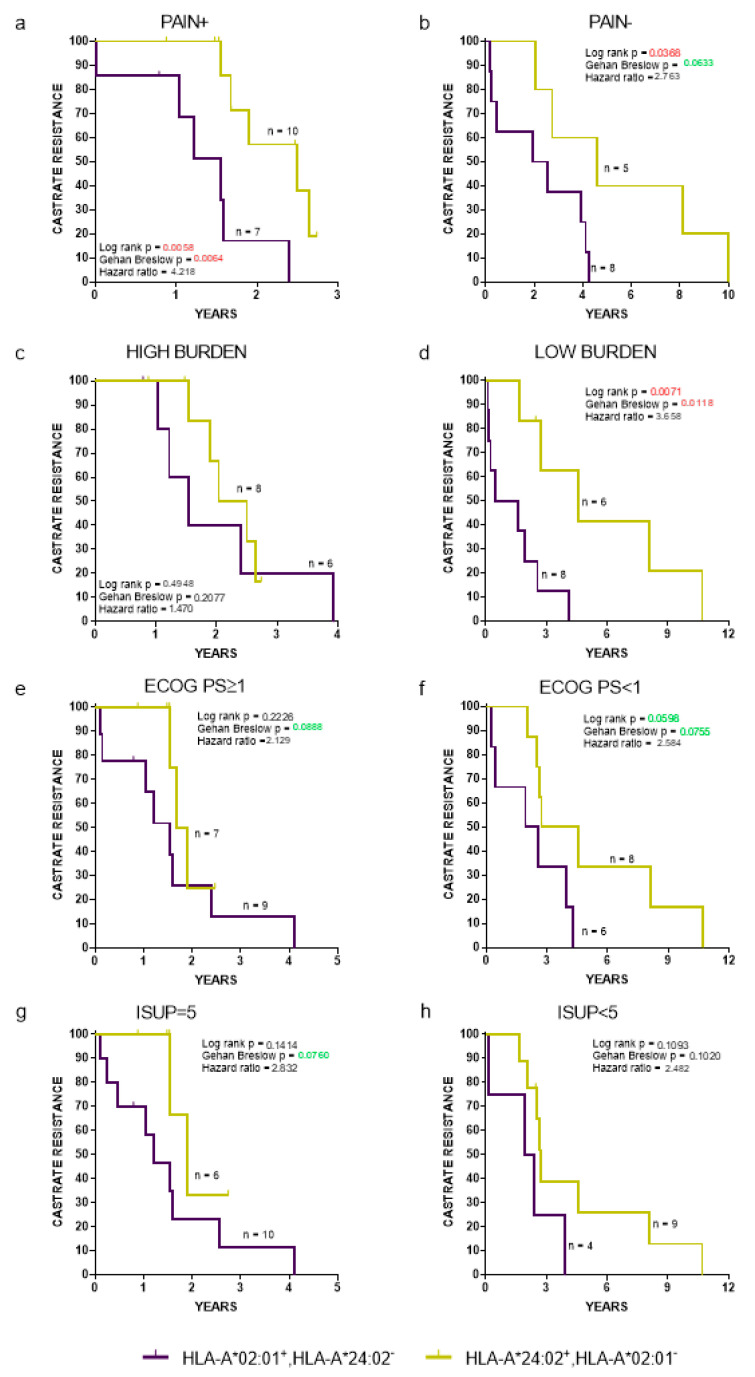
Kaplan-Meier curves illustrate time to CR progression for HLA-A*02:01^+^HLA-A*24:02^−^ vs. A*24:02^+^HLA-A*202:01^−^ patients, stratified by presence (**a**) or absence (**b**) of pain; high (**c**) or low (**d**) tumor burden; Eastern cooperative oncology group performance status ≥1 (**e**) or <1 (**f**); International Society of Urological Pathology = 5 (**g**) or <5 (**h**). ECOG PS: Eastern cooperative oncology group performance status and ISUP: International Society of Urological Pathology.

Clinicopathological parameters related with de novo mPCa patient prognosis include pain (presence or absence), Eastern cooperative oncology group performance status (ECOG PS) (<1 vs. ≥1), International Society of Urological Pathology (ISUP), grade of PCa (<5 vs. 5), and disease burden (high vs. low) [16,27]. A high burden was defined as the presence of visceral metastases or four or more bone metastases with >1 bone lesions beyond the pelvis or axis [16]. The time to the progression to CR (Figure 3) and OS (Figure S1), regardless all the aforementioned clinicopathological parameters, was HLA-dependent, with HLA-A*24:02^−^HLA-A*02:01^+^ patients exhibiting strong trends for a worse clinical outcome, reaching, in some cases, statistical significance compared to the corresponding HLA-A*24:02^+^HLA-A*202:01^−^ patient cohort.

### 2.4. Clinical Outcomes in HLA-A*02:01 Homozygous vs. Heterozygous Patients

**Figure 4:**
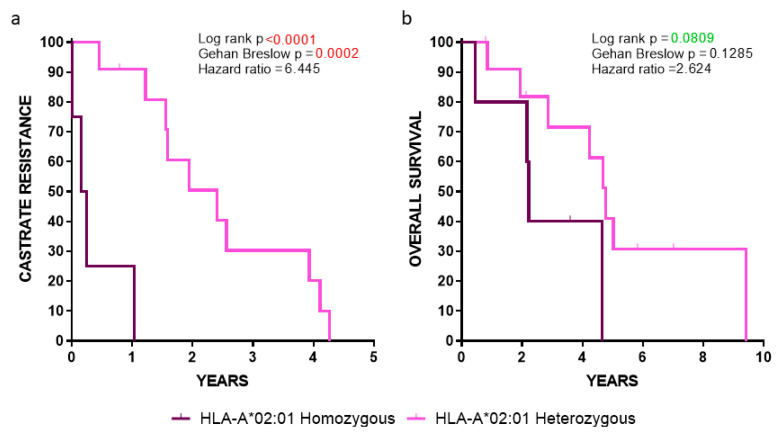
Kaplan-Meier curves illustrating the time to (**a**) CR and (**b**) OS in the indicated groups of patients.

Our analyses showed a robust statistical significance among HLA-A*02:01 homozygous vs. heterozygous patients, with the latter group (lacking the HLA-A*24:02 allele) progressing much slower to CR (*p* < 0.0001) (Figure 4a), whereas a strong trend was observed between these two groups when the OS was the clinical endpoint (*p* = 0.0809) (Figure 4b). Given that these statistical values were obtained despite the low number of HLA-A*02:01 homozygous patients (*n* = 5; median survival 2.19 years) vs. the heterozygous HLA-A*02:01 ones lacking the HLA-A*24:02 allele, (*n* = 12; median survival 4.45 years), our data emphasized the key role of HLA-A*02:01 as a poor prognosticator.

### 2.5. HLA Status as an Independent Prognostic Biomarker

To investigate the prognostic significance of the HLA-A*02:01 vs. HLA-A*24:02 expression in the absence of HLA-A*24:02 or HLA-A*02:01, respectively, and irrespective of the second HLA class I allele, we conducted univariate and multivariate analyses using as covariates the established clinicopathological factors [12,16,27]. For de novo mPCa, with CR and OS as the endpoints, we examined, as covariates, age, PSA, ISUP grade group, disease burden, localization of the metastases, pain, ECOG PS, and HLA allele expression.

In the univariate analyses (Table 1), a statistically strong negative impact for HLA-A*02:01 in the absence of HLA-A*24:02 (*p* = 0.007) or for HLA-A*02:01 irrespective of the second HLA-allele (*p* = 0.048) on the progression to CR was revealed, which was weaker when the OS was considered as the endpoint (*p* = 0.065 and *p* = 0.076, respectively) (Table 1 and Table S1). After the stepwise selection in the multivariate analysis (Table 1), the expression of the HLA-A*02:01 allele, in the absence of HLA-A*24:02 and vice versa, was found (i) to be the strongest prognostic factor for the progression to CR (*p* = 0.001) and (ii) a strong prognosticator for the OS (*p* = 0.016) (in both cases, along with pain). The results were similar even when evaluating the HLA-A*02:01 expression without taking into consideration the HLA-A*24:02 expression (Table S1).

## 3. Discussion

In the present study, we demonstrated that de novo mPCa patients expressing the HLA-A*02:01 allele exhibited poor clinical outcomes (CR and OS) compared to their HLA-A*202:01^−^negative counterparts. Furthermore, we presented strong evidence that HLA-A*24:02 has an impact on disease progression, enabling favorable clinical outcomes. Our data demonstrated, for the first time, an interrelationship between these two HLA class I alleles, in that HLA-A*02:01 in the absence of HLA-A*24:02 and vice versa have significant opposing roles as independent bad or good prognosticators, respectively, for de novo mPCa.

Metastatic disease may be presented de novo at the initial diagnosis or as a progression following a definitive therapy for localized PCa. De novo mPCa defines a limited (approximately 4% of PCa) subset of patients with aggressive disease and unfavorable clinical outcomes compared to patients who developed metastases after a curative treatment [5]. Despite the excessive clinical and laboratory research regarding progressed mPCa, only a limited number of studies to date have focused on de novo mPCa, with mainly retrospective analyses on clinical outcomes [17,28]. Appropriate treatment modalities for de novo mPCa have not yet been established, as indicated by the recent [29,30] and ongoing clinical trials [31] (https://clinicaltrials.gov).

Given the pivotal role ascribed to major histocompatibility complex (MHC) gene products as the orchestrators of immune responses, it is not surprising that these genes have an important influence on cancer development [32]. HLA class I abnormalities were reported to correlate with the clinical outcomes of PCa [33,34]. However, these abnormalities are not directly related with specific HLA alleles, but they are rather linked to abnormalities in the expression of various components belonging to the MHC class I-related antigen-processing and presentation pathways. Factors implicated in the downregulation of HLA expression include several oncogenes (e.g., HER-2/neu, RAS, and MYC) [35] also related to the transition of hormone-sensitive to castrate-resistant PCa [36].

The HLA-A2 phenotype, which is overrepresented among Swedish castration-resistant PCa (CR-PCa) patients, was suggested, though without clear confirmation, to associate with high mortality rates, acting either as a risk factor or as a poor prognosticator [23]. Albeit at similar lines with our results, still these data may be considered only as indicative, given that (i) the number of CR-PCa patients analyzed was very small, and (ii) the HLA-A2 phenotype was defined with a monoclonal antibody also recognizing HLA-B*57/B*58 (expressed by about 5% of the Swedish population) and not discriminating among the HLA-A2 sub-alleles (e.g., A*02:01 from others). Furthermore, similar assumptions were made for the higher mortality among Swedish ovarian cancer patients [23]. Nevertheless, analyses from this study were performed during therapies, and thus, it was obscure whether the segregation of patients carrying this allele was a result of therapeutic treatments or its expression influenced the natural development of the disease independent of therapies. Moreover, in a subsequent study, it was shown that an increased mortality among patients with ovarian carcinoma was rather due to a downregulation of HLA class I expression caused by HER-2/neu overexpression and/or haplotype loss [37], mechanisms not specifically related to the HLA-A2 phenotype.

On the other hand, our findings do not support the hypothesis for increased PCa incidences in HLA-A2^+^ patients: among 187 PCa patients, the HLA-A*02:01 phenotype frequency was decreased compared to the general Greek population (36.36% vs. ≈43%), whereby the HLA-A*24:02 was overrepresented (36.36% vs. ≈22%) (unpublished data including the 56 de novo mPCa patients of the current study), thus disputing the hypothesis of HLA-A2 as a risk factor for PCa. Our data also contradicted a possible favorable prognostic role of HLA-A2 as proposed by De Petris et al. [23], who hypothesized, though without clinical evidence, that most CR-PCa patients expressing this allele survived to receive repeated lines of therapy. On the contrary, in our study, HLA-A*02:01^+^ de novo mPCa patients progress significantly earlier to CR and also had shorter OS as compared their HLA-A*202:01^−^ counterparts.

The underlying etiology for the role of HLA-A*02:01 on the worse prognosis might be attributed either to a selective sub-allele expression loss (e.g., selective gene loss, chromosome deletion/translocation, or mutation) and/or immune pressure for the selection of tumor cell clones not, or elusively, recognized through HLA-A*02:01 as a result of the heterogenic expression of tumor peptides specifically restricted by this allele. This may also explain the more severe clinical outcomes in patients being homozygous for HLA- A*02:01. In addition, HLA-A2 mutations leading to the allele expression loss have been sporadically reported in the past in melanoma and cervical carcinoma cell lines by standard PCR and sequencing techniques [38,39,40]. The detection of somatic mutations in HLA genes using whole-exome sequencing by high-throughput techniques and the interpretation of these remains challenging due to the high polymorphism of the HLA loci [41,42]. This might explain why such mutations have not been detectable and reported so far in large genomic analyses in PCa.

Conversely, the favorable role of HLA-A*24:02 expression on PCa clinical outcomes might be interpreted by the increased immunogenicity of HLA-A24^+^ PCa cells, leading to long periods of tumor control under immunosurveillance during “equilibrium”. Extended analyses on a greater cohort of patients are surely needed to corroborate these hypotheses; in which case, the underlying mechanisms ascribing an “unfavorable” or “favorable” prognostic role in HLA class I alleles should be explored.

There is an unmet need to identify prognostic factors in order to improve the clinical decision-making process for PCa. Different variables are combined in biologically and clinically integrated models in order to develop nomograms/biomarkers for a more personalized approach in cancer [43,44]. However, few studies have been performed for the development of novel nomograms in de novo mPCa [45]. To this end, the GS/ISUP grade group, PCa burden, ECOG PS, and pain [16,27] are being used in the clinical praxis for the prognosis of de novo metastatic PCa despite the fact that these have been described as insufficient for accurately stratifying heterogenous tumors [46]. Our findings propose, for this particular group of patients, the HLA-A*02:01 and HLA-A*24:02 alleles as potential markers of interest in predicting the disease progression to CR and patient survival. More importantly, our data may also provide a rationale to account for a possible prevalence of the HLA phenotype over clinicopathologic criteria to forecast which tumors have a high risk of recurrence. In view of our results, it may be intriguing to include, in the future, HLA alleles as prognostic factors in evaluating the prognosis in patients with de novo metastatic PCa.

The data reported here also clearly demonstrated the unfavorable prognostic potential of HLA-A*02:01 expression, especially in homozygosity. Thus, it is reasonable to suggest that this particular HLA phenotype might be incorporated in the prognostic de novo mPCa algorithms, as evidenced by our multivariate analysis. This would contribute to better therapeutic interventions, leading to intensified treatment modalities in high-risk patients or avoiding the overtreatment of lower risk ones. Thus, HLA-A locus typing at diagnosis would be of importance for this relatively rare group of PCa patients, although relatively costly as a molecular prognostic test. However, the ease with which HLA-A*02:01 can be detected, and with low cost, by routine flow cytometry, not requiring the more expensive HLA-typing next generation sequencing technology, further supports its use as a prognostic factor.

The present study has limitations, first due to the relatively small cohort of patients evaluated, (mostly due to the rarity of this PCa subgroup) and, second, due to the fact that all patients were diagnosed and treated in a single oncology hospital in Athens. Thus, further analyses are required in multicenter international studies including larger cohorts of de novo mPCa patients. Furthermore, the possibility of the prognostic potential of these two alleles in patients with localized PCa should be also investigated, as well as the underlying mechanisms explaining the differential roles of different alleles in PCa development and progression.

## 4. Materials and Methods

### 4.1. Study Population and Study Design

**Scheme 1:**
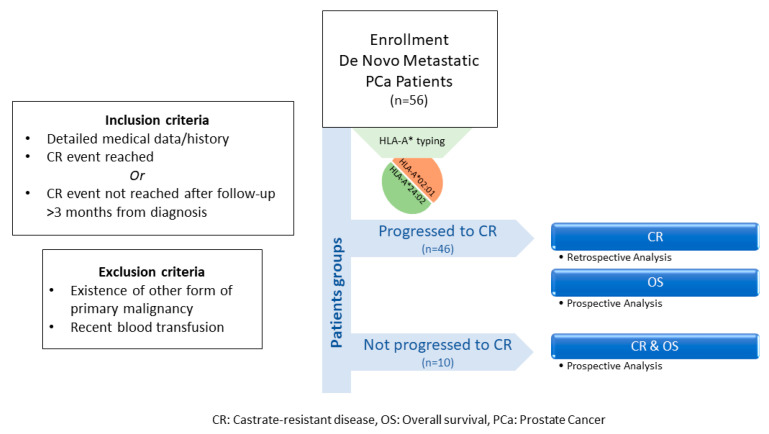
Flowchart of study design.

Medical records of 56 de novo mPCa patients from the “Saint Savas Cancer Hospital” in Greece were reviewed between 3/2017–12/2019. Written informed consent was obtained from all patients enrolled. The study and the informed consent forms were approved by the Hospital IRB (IRB-ID6777/14-06-2017) and the Ethical Committee of the University of Athens (IRB-ID1516015872/03-02-2016). Patients enrolled in this combined retrospective/prospective study first diagnosed between 2004-2019 received standard medical treatment upon diagnosis and had complete medical records, including baseline disease characteristics, treatments received, and clinical follow-up before and after enrollment. Eligible patients either had progressed to CR or had a clinical follow-up >3 months from diagnosis with no progression to CR [47]. Patients who had progressed to CR, were analyzed retrospectively for the TTP (time to progression) to CR and prospectively for OS, whereas those with no progression to CR were analyzed for both CR and OS, prospectively (see also Scheme 1). Patients with other primary malignancies, or with a recent blood transfusion, were excluded. Blood for HLA class I typing was collected at the time of enrollment. Patients were prospectively followed-up at scheduled timepoints. The endpoints of the study were (i) the time to CR from the initiation of the androgen deprivation therapy and (ii) OS from diagnosis. Clinical evaluation was assessed according to the Response Evaluation Criteria In Solid Tumors (RECIST) (Version 1.1) [48]. Abdominal CT scan with contrast and/or whole-body bone scan were performed for monitoring progression. CR was defined as castrate serum testosterone <50 ng/dL plus biochemical (three consecutive rises in PSA 1 week apart, resulting in two 50% increases over the nadir, and PSA > 2 ng/mL) or radiologic progression (the appearance of new lesions).

**Figure 5:**
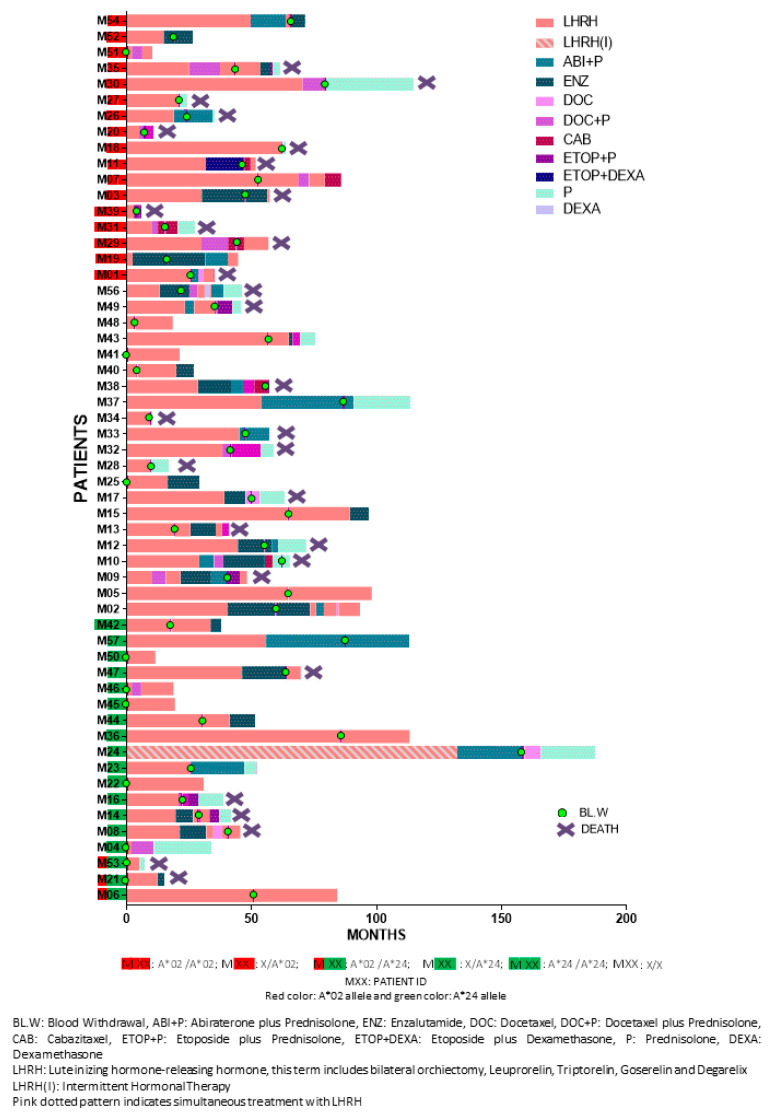
Individual treatment sequence and human leukocyte antigen (HLA)-A expression.

The clinicopathological characteristics of the enrolled patients are presented in Table 2. Median follow-up period was 3.76 years (range: 0.32–15.35). Detailed patient information is provided in Table S2. Figure 5 depicts the detailed sequence and timepoint or period for each intervention per patient. Study design is schematically summarized in Scheme 1.

### 4.2. HLA Typing

HLA class I antigen genotyping was performed using next generation sequencing for the A-locus (ONE LAMBDA Inc, Los Angeles, CA, USA and Thermo Fisher Scientific, Waltham, MA, USA).

### 4.3. Statistical Analysis

GraphPad Prism v.8.0 software was used for cumulative survival probabilities testing using the Kaplan-Meier analysis with 95% confidence intervals (95% CIs) to evaluate the possible association of HLA expression with the clinical outcome. Survival curves were calculated and compared using the log-rank test (Mantel-Cox), the Gehan-Breslow Wilcoxon test, and mentioning the hazard ratio (HR; log rank). Statistical differences were considered significant for *p*-values < 0.05. Values between 0.1–0.05 were considered as a trend. Univariate and multivariate survival analyses (Cox regression) were conducted by IBM SPSS 24. For the multivariate analysis, the forward stepwise method with a threshold of 0.05 as an entry point was used. 

## 5. Conclusions

We documented unfavorable and favorable prognostic impacts of HLA alleles, namely HLA-A*02:01 and HLA-A*24:02, respectively, in de novo mPCa. These alleles, as independent biomarkers, along with established prognostic criteria, might improve the appropriate treatment modalities selection and avoid overtreatment. The possibility that these alleles are also related to the clinical outcome of the vast majority of PCa patients, i.e., those initially diagnosed with a localized disease, should also be evaluated. The possible positive or negative impacts of other HLA alleles underrepresented in the Greek population might also be investigated. However, it is becoming obvious that, apart from their crucial role on the overall antigen-presentation machinery, individual HLA alleles differentially affect PCa evolution and progression, although the underlying mechanisms remain unclear.

## Figures and Tables

**Table 1:** Univariate analysis and multivariate analysis of risk factors.

**Univariate**	**CR**			**OS**		
	***p***	**Hazard Ratio**	**95.0% CI for Exp(B) (Range)**	***p***	**Hazard Ratio**	**95.0% CI for Exp(B) (Range)**
**Age**	0.978	0.992	0.573–1.719	0.701	0.888	0.483–1.630
**PSA**	0.495	1.000	0.999–1.001	0.798	1.000	0.999–1.001
**ISUP Grade group**	0.181	1.215	0.913–1.6177	0.034	1.507	1.032–2.200
**PCa burden**	0.258	1.393	0.784–2.473	0.822	1.077	0.565–2.052
**Metastases**	0.955	1.011	0.690–1.481	0.777	0.940	0.615–1.438
**Pain**	0.005	2.333	1.286–4.232	0.000	3.937	1.964–7.893
**ECOG PS**	0.003	2.433	1.346–4.397	0.003	2.745	1.418–5.314
**HLA-A allele**	0.007	1.675	1.150–2.438	0.065	1.521	0.975–2.372
**Multivariate**	**CR**			**OS**		
	***p***	**Hazard Ratio**	**95.0% CI for Exp(B) (Range)**	***p***	**Hazard Ratio**	**95.0% CI for Exp(B) (Range)**
Model before Stepwise Selection						
**Age**	0.651	1.181	0.574–2.431	0.347	1.440	0.673–3.079
**PSA**	0.914	1.000	0.999–1.001	0.404	1.001	0.999–1.002
**ISUP Grade group**	0.351	1.140	0.865–1.501	0.103	1.332	0.943–1.882
**PCa burden**	0.944	0.967	0.374–2.499	0.986	1.008	0.423–2.399
**Metastases**	0.654	1.124	0.674–1.875	0.910	1.033	0.586–1.823
**Pain**	0.140	2.333	0.757–7.190	0.006	1.889	1.544–13.399
**ECOG PS**	0.978	1.012	0.428–2.395	0.597	0.777	0.298–2.007
**HLA-A allele**	0.008	2.029	1.205–3.414	0.051	2.035	0.998–4.150
Model after Stepwise Selection						
**HLA-A allele**	0.001	2.052	1.355–3.108	0.016	1.889	1.126–3.169
**Pain**	0.013	2.222	1.181–4.179	0.002	3.116	1.512–6.424

CR: castration–resistance; OS: overall survival; CI: confidence interval; PSA: prostate–specific antigen; ISUP: International Society of Urological Pathology; ECOG PS: Eastern cooperative oncology group performance status; Exp: exponentiation; GS: Gleason Score. All categorical covariates were transformed into numeric codes as follows: age: <70, 1 and ≥70, 2; ISUP grade group: GS ≤6, 1; 3+4, 2; 4+3, 3; 8, 4; and ≥9, 5; PCa burden: low, 1 and high, 2; metastases: only lymph node, 1; only bone, 2; and lymph node and bone, 3; pain: absent, 1 and present, 2; ECOG PS: negative, 1; ≥1, 2; and HLA-A allele: HLA-A*24:02^+^HLA-A*202:01^−^, 1; rest, 2; and HLA-A*02:01^+^HLA-A*24:02^−^, 3.

**Table 2:** Clinicopathological Characteristics of patients.

**PCa Patients with de novo metastatic disease**	
Characteristic (Initial PCa Staging)	No. (%) of PCa (*N* = 56)
**Median age at diagnosis (years) (range)**	70.14 (49–88%)
**PSA (median 75.9 ng/mL; range 2.38–6806)**	
≤100	30 (53.6%)
>100	23 (41.1%)
Missing	3 (5.4%)
**Gleason Score**	
≤7	10 (17.9%)
8	14 (25%)
≥9	27 (48.2%)
Missing	5 (8.9%)
**ISUP Grade group**	
1	2 (3.6%)
2	2 (3.6%)
3	6 (10.7%)
4	14 (25%)
5	27 (48.2%)
Missing	5 (8.9%)
**Nodal Status ^a^**	
Positive	29 (51.9%)
Negative	24 (42.9%)
Missing	3 (5.4%)
**Bone Metastasis ^b^**	
Positive	46 (82.1%)
Negative	7 (12.5%)
Missing	3 (5.4%)
**High-Volume PCa (visceral metastases and/or 4 or more bone metastases)**	
Positive	28 (50%)
Negative	25 (44.6%)
Missing	3 (5%)
**ECOG PS**	
0	31 (55.4%)
≥1	25 (44.6%)
**PAIN**	
Present	33 (58.9%)
Absent	23 (41.1%)
**Ethnicity**	
Caucasian	54 (96.4%)
African American	1 (1.8%)
Asian	1 (1.8%)

^a^ As reported by Computed Tomography (CT) scan (abdominal). ^b^ As defined by Whole Body Bone Scan (WBS).

## Supplementary Materials

The following are available online at https://www.mdpi.com/2072-6694/12/6/1623/s1: Table S1. Univariate analysis and multivariate analysis of risk factors. Table S2. Individual clinicopathological characteristics, treatment sequence, clinical outcome, and HLA-A expression. Figure S1. Kaplan-Meier curves illustrate time to OS for HLA-A*02:01^+^HLA-A*24:02^−^ vs. HLA-A*24:02^+^HLA-A*02:01^−^ patients stratified by the indicated clinicopathological criteria.
