# Supplementary material for: HLA Class I Allele Expression and Clinical Outcome in De Novo Metastatic Prostate Cancer
Source: Cancers (Basel). 2020 Jun 18;12(6):1623. doi: 10.3390/cancers12061623 (PMC7352811; doi:10.3390/cancers12061623)
Supplement: Supplementary file 1 [file cancers-12-01623-s001.pdf]

SupplementaryMaterials:

HLA Class I Allele Expression and Clinical Outcome in De Novo Metastatic Prostate Cancer

Savvas Stokidis, Sotirios P. Fortis, Paraskevi Kogionou, Theodoros Anagnostou, Sonia A. Perez and Constantin N. Baxevanis

Table S1: Univariate Analysis and multivariate Analysis of risk factors.

Univariate	CR			OS		
	**p**	**Hazard Ratio**	**95.0% CI for Exp(B) (Range)**	**p**	**Hazard Ratio**	**95.0% CI for Exp(B) (Range)**
**Age**	0.978	0.992	0.573–1.719	0.701	0.888	0.483–1.630
**PSA**	0.431	0.878	0.636–1.213	0.188	0.787	0.550–1.124
**ISUP Grade group**	0.181	1.215	0.913–1.6177	0.034	1.507	1.032–2.200
**PCa burden**	0.258	1.393	0.784–2.473	0.822	1.077	0.565–2.052
**Metastases**	0.955	1.011	0.690–1.481	0.777	0.940	0.615–1.438
**Pain**	0.005	2.333	1.286–4.232	0.000	3.937	1.964–7.893
**ECOG PS**	0.003	2.433	1.346–4.397	0.003	2.745	1.418–5.314
**HLA-A allele**	0.048	1.747	1.004–3.039	0.076	1.762	0.942–3.295
**Multivariate**	**CR**			**OS**		
	**p**	**Hazard Ratio**	**95.0% CI for Exp(B) (Range)**	**p**	**Hazard Ratio**	**95.0% CI for Exp(B) (Range)**
Model before Stepwise Selection						
**Age**	0.562	1.239	0.600–2.558	0.260	1.567	0.717–3.427
**PSA**	0.388	.999	0.998–1.001	0.438	1.000	0.999–1.002
**ISUP Grade group**	0.234	1.187	0.895–1.574	0.63	1.389	0.982–1.966
**PCa burden**	0.804	1.127	0.439–2.896	0.733	1.187	0.480–2.837
**Metastases**	0.649	1.126	0.675–1.880	0.656	1.135	0.650–1.983
**Pain**	0.213	2.048	0.663–6.323	0.005	5.200	1.659–16.301
**ECOG PS**	0.899	1.059	0.433–2.593	0.379	0.654	0.253–1.687
**HLA-A allele**	0.015	2.636	1.206–5.762	0.005	3.675	1.470–9.189
Model after Stepwise Selection						
**HLA-A allele**	0.002	2.595	1.401–4.806	0.003	2.863	1.417–5.785
**Pain**	0.011	2.327	1.212–4.466	0.001	3.558	1.683–7.520

All categorical covariates were transformed into numeric codes as follows: Age: <70; 1, ≥70; 2, PSA: ≤50; 1, >50-≥100; 2, >100; 3, ISUP grade group: GS ≤6; 1, 3+4; 2, 4+3; 3, 8; 4, ≥9; 5, PCa burden: low; 1, high; 2, Metastases: only Lymph Node; 1, only Bone; 2, Lymph Node and Bone; 3, Pain: absent; 1, present; 2, ECOG PS: negative; 1, ≥1; 2 & HLA-A allele: HLA-A*02:01^{-}; 1, HLA-A*02:01^{+}; 2.

Table S2. Individual clinicopathological characteristics, treatment sequence, clinical outcome, and HLA-A expression.

Patient	Age (years)	Baseline PSA (ng/mL)	Gleason Score	All disease sites at baseline◊	Burden PCa (High: visceral metastases and/or 4 or more bone metastases)	ECOG PS	Pain	Treatments	Endpoints		HLA-A*	
									Time from diagnosis to CRPC	OS (from diagnosis) (years)		
M01	81	5.79	10 (5+5)	BONE	HIGH	≥1	PRESENT	BIC,LEU,ABI+P,DOC,CAB	1.04	2.16	HLA-A*02:01	HLA-A*02:01
M02	66	18.7	9 (4+5)	BONE	HIGH	0	ABSENT	DEG,BIC,ENZ,ABI+P,DOC	2.99	(7.64)	HLA-A*26:01	HLA-A*66:01
M03	82	71.32	7 (3+4)	BONE	HIGH	≥1	PRESENT	DEG,BIC,ENZ,P	2.40	4.67	HLA-A*02:01	HLA-A*68:01
M04	52	738.6	9 (4+5)	LN,BONE	HIGH	0	PRESENT	DEG,DOC,P	(2.74)	(2.74)	HLA-A*32:01	**HLA-A*24:02**
M05	65	47.13	8 (4+4)	LN,BONE	LOW	0	ABSENT	DEG	(8.01)	(8.01)	**HLA-A*02:05**	HLA-A*32:01
M06	56	617.9	8 (4+4)	LN,BONE	LOW	0	ABSENT	DEG	(6.87)	(6.87)	HLA-A*02:01	**HLA-A*24:02**
M07	73	243.7	9 (4+5)	LN	LOW	0	ABSENT	BIC,LEU,P,DOC,CAB	2.56	(7.01)	HLA-A*02:01	HLA-A*26:01
M08	76	119.2	8 (4+4)	BONE	LOW	≥1	PRESENT	BIC,LEU,ENZ,DOC	1.68	3.72	HLA-A*26:01	**HLA-A*24:02**
M09	66	1000	9 (4+5)	BONE	HIGH	≥1	PRESENT	BIC,TRIPT,DOC,P,LEU,ENZ,ABI+P,ETOP	0.16	3.93	HLA-A*30:01	HLA-A*33:01
M10	66	789.1	8 (4+4)	BONE	HIGH	0	ABSENT	DEG,BIC,ABI+P,DOC, ABI+P,ENZ,CAB	1.77	5.38	HLA-A*03:01	HLA-A*68:01
M11	64	16.11	9 (4+5)	LN,BONE	LOW	≥1	PRESENT	DEG,LEU,ETOP+DEXA,CAB	1.59	4.22	HLA-A*02:01	HLA-A*30:01
M12	81	41.9	7 (4+3)	LN,BONE	LOW	≥1	ABSENT	DEG,BIC,ENZ,ABI+P	3.66	5.87	HLA-A*01:01	HLA-A*26:01
M13	70	68.06	7 (4+3)	LN,BONE	HIGH	0	PRESENT	BIC,LEU,ENZ,DOC	1.95	3.33	HLA-A*32:01	HLA-A*11:01
M14	75	1356	9 (4+5)	BONE	HIGH	≥1	PRESENT	DEG,ENZ,DOC,P,ETOP	1.55	3.41	HLA-A*01:01	**HLA-A*24:02**
M15	80	24.88	10 (5+5)	BONE	LOW	0	ABSENT	BIC,TRIPT,ENZ	7.31	(7.92)	HLA-A*03:02	HLA-A*11:01

M16	61	59.6	8 (4+4)	BONE	HIGH	0	PRESENT	BIC,LEU,DOC,ETOP	2.50	3.17	HLA-A*33:03	**HLA-A*24:02**
M17	71	360.26	8 (4+4)	LN,BONE	LOW	≥1	PRESENT	BIC,LEU,ENZ,DOC	1.50	5.17	HLA-A*01:01	HLA-A*03:01
M18	75	497.6	9 (4+5)	LN,BONE	LOW	≥1	ABSENT	BIC,LEU,P	4.11	4.78	HLA-A*02:01	HLA-A*01:01
M19	84	104	8 (4+4)	BONE	LOW	≥1	ABSENT	LEU,ENZ,ABI+P	0.15	(3.59)	HLA-A*02:01	HLA-A*02:01
M20	73	58	9 (5+4)	LN,BONE	LOW	0	ABSENT	BIC,TRIPT,DOC,P	0.46	0.85	HLA-A*02:01	HLA-A*26:01
M21	65	1303	6 (3+3)	LN,BONE	HIGH	0	PRESENT	DEG,ENZ	0.71	1.21	HLA-A*02:01	**HLA-A*24:02**
M22	86	124	8 (4+4)	LN	LOW	≥1	PRESENT	DEG	(2.47)	(2.47)	HLA-A*32:01	**HLA-A*24:02**
M23	70	79.3	9 (4+5)	BONE	HIGH	≥1	PRESENT	BIC,LEU,ABI+P	1.90	(4.49)	HLA-A*30:02	**HLA-A*24:02**
M24	59	75.9	8 (4+4)	BONE	LOW	0	ABSENT	LEU,ABI+P,DOC	10.70	(15.35)	HLA-A*26:01	**HLA-A*24:02**
M25	69	1000	MISSING	BONE	HIGH	≥1	PRESENT	DEG,ENZ	2.02	(3.05)	HLA-A*03:01	HLA-A*68:01
M26	49	21	9 (4+5)	BONE	HIGH	≥1	PRESENT	DEG,ABI+P	1.55	2.87	HLA-A*02:01	HLA-A*32:01
M27	84	14.88	9 (4+5)	LN	LOW	0	PRESENT	DEG,P	ND	1.95	HLA-A*02:01	HLA-A*25:01
M28	71	4.1	MISSING	LN,BONE	LOW	≥1	PRESENT	LEU	0.84	1.36	HLA-A*01:01	HLA-A*32:01
M29	68	20	9 (5+4)	BONE	LOW	≥1	PRESENT	LEU,DOC+P,CAB	ND	4.64	HLA-A*02:01	HLA-A*02:01
M30	73	MISSING	9 (5+4)	MISSING	MISSING	0	ABSENT	DEG,DOC+P	4.26	9.41	HLA-A*02:01	HLA-A*23:01
M31	64	10.01	10 (5+5)	LN	LOW	≥1	PRESENT	LEU,DOC+P,CAB	0.00	2.22	HLA-A*02:01	HLA-A*02:01
M32	68	35	8 (4+4)	BONE	HIGH	0	PRESENT	TRIPT,DOC+P	ND	3.99	HLA-A*01:01	HLA-A*69:01
M33	80	11	8 (4+4)	MISSING	MISSING	0	ABSENT	DEG,ABI+P	ND	4.67	HLA-A*01:01	HLA-A*03:01

M34	67	342	MISSING	LN,BONE	HIGH	≥1	PRESENT	LEU	0.58	0.80	HLA-A*26:01	HLA-A*03:01
M35	66	38.26	8 (4+4)	LN	LOW	0	ABSENT	GOS.BIC,DOC+P,DEXA,ENZ,ETOP+P	1.94	5.02	HLA-A*02:01	HLA-A*26:01
M36	61	34.77	7 (3+4)	BONE	LOW	0	ABSENT	LEU,BIC	8.10	(9.25)	HLA-A*33:01	**HLA-A*24:02**
M37	69	183	6 (3+3)	BONE	HIGH	0	PRESENT	DEG,BIC,ABI+P	3.44	(9.29)	HLA-A*03:01	HLA-A*68:01
M38	80	134.3	9 (4+5)	LN,BONE	LOW	0	ABSENT	BIC,LEU,DEG,ENZ,ABI+P,DOC,CAB	1.19	4.67	HLA-A*03:01	HLA-A*03:01
M39	56	9	9 (4+5)	LN,BONE	LOW	0	ABSENT	LEU,ETOP	0.24	0.46	HLA-A*02:01	HLA-A*02:01
M40	65	100	7 (4+3)	LN,BONE	HIGH	0	PRESENT	DEG,BIC,ENZ	1.60	(2.15)	HLA-A*11:01	**HLA-A*24:07**
M41	78	388.4	9 (5+4)	BONE	LOW	0	ABSENT	BIC,LEU	(1.70)	(1.70)	HLA-A*33:03	HLA-A*03:01
M42	71	126.73	8 (4+4)	LN,BONE	HIGH	0	PRESENT	DEG,LEU,ENZ	2.65	(3.01)	**HLA-A*24:02**	**HLA-A*24:02**
M43	49	73	9 (4+5)	LN,BONE	HIGH	0	PRESENT	LEU,ENZ,DOC	5.27	(6.13)	HLA-A*02:02	HLA-A*23:01
M44	67	70.11	7 (4+3)	LN,BONE	HIGH	0	ABSENT	BIC,LEU,ENZ	2.04	(4.20)	HLA-A*29:01	**HLA-A*24:02**
M45	81	MISSING	9 (5+4)	MISSING	MISSING	≥1	PRESENT	LEU	(1.53)	(1.53)	HLA-A*03:01	**HLA-A*24:02**
M46	70	841	9 (4+5)	LN,BONE	HIGH	≥1	PRESENT	LEU,DOC	(1.48)	(1.48)	HLA-A*03:01	**HLA-A*24:02**
M47	88	5.5	8 (4+4)	BONE	LOW	0	ABSENT	BIC,LEU,ENZ	2.73	4.71	**HLA-A*24:02**	HLA-A*26:01
M48	84	6806	MISSING	BONE	HIGH	≥1	PRESENT	BIC,LEU,TRIPT	(1.47)	(1.47)	HLA-A*01:01	HLA-A*68:01
M49	65	2.38	9	BONE	HIGH	0	PRESENT	LEU,ABI+P,ETOP	0.00	3.75	HLA-A*03:01	HLA-A*68:01
M50	88	740,3	9 (5+4)	BONE	HIGH	≥1	PRESENT	LEU	(0.88)	(0.88)	HLA-A*01:01	**HLA-A*24:02**
M51	64	183.6	9 (5+4)	BONE	HIGH	≥1	PRESENT	LEU,DOC+P	(0.79)	(0.79)	HLA-A*02:01	HLA-A*01:01

M52	67	7	10 (5+5)	LN,BONE	HIGH	≥1	PRESENT	LEU,ENZ,DOC	1.22	(2.13)	HLA-A*02:01 HLA-A*30:01
M53	68	291.5	10 (5+5)	LN,BONE	HIGH	≥1	PRESENT	LEU,DEXA	(0.32)	0.32	HLA-A*02:01 **HLA-A*24:02**
M54	80	MISSING	7 (4+3)	LN,BONE	HIGH	0	ABSENT	BIC,LEU,ABI+P,ENZ	3.93	(5.82)	HLA-A*02:01 HLA-A*32:01
M56	71	81.72	9 (4+5)	LN	LOW	0	ABSENT	BIC,LEU,SRTX,ENZ,DOC,DEXA,ABI+P	1.04	3.78	HLA-A*01:01 HLA-A*31:01
M57	84	14	7 (4+3)	BONE	LOW	0	ABSENT	BIC,DEXA,TRIPT,ABI+P	4.57	(9.13)	HLA-A*01:01 **HLA-A*24:02**

ABI: Abiraterone, ABI+P: Abiraterone plus Prednisolone, CAB: Cabazitaxel, CARBO: Carboplatine, DEG: Degarelix, DEXA: Dexamethasone, DOC: Docetaxel, DOC+P: Docetaxel plus Prednisolone, ENZ: Enzalutamide, ETOP: Etoposide, ETOP+DEXA: Etoposide plus Dexamethasone, ETOP+P: Etoposide plus Prednisolone, GOS: Goserelin, LEU: Leuprorelin, LN: lymph nodes, P: Prednisolone, TRIPT: Triptorelin. ◊As reported by CT scan (abdominal) or bone scan; ND = Not Determined; Parentheses indicate censoring of the data at the last time the patient was known not to present the event.

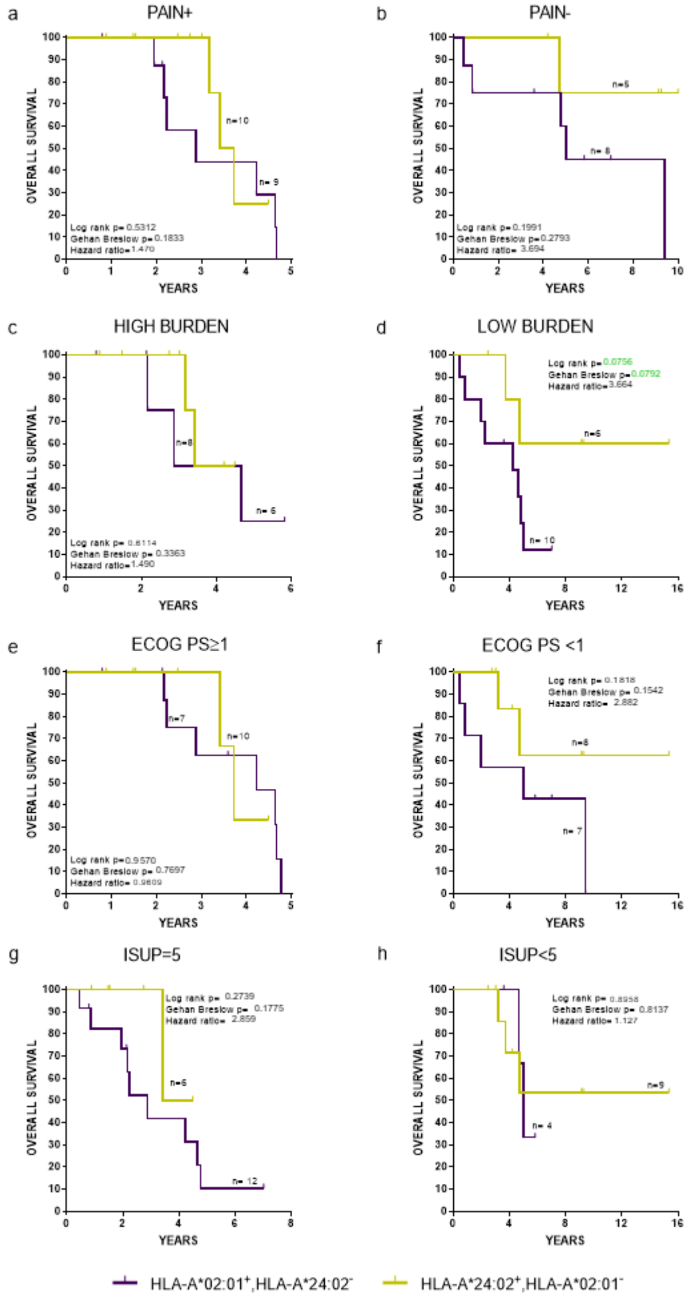

Figure S1. Kaplan-Meier curves illustrate time to OS for HLA-A*02:01^{+}HLA-A*24:02^{-} vs. HLA-A*24:02^{+}HLA-A*02:01^{-} patients, stratified by the indicated clinicopathological criteria.
